# The Development of a Scale to Measure HIV-Prevention Factors in Adults Age 50 and Older

**DOI:** 10.1093/geroni/igaa057.1614

**Published:** 2020-12-16

**Authors:** Rachel Wion, Susan Loeb, Jacqueline Mogle, Donna Fick

**Affiliations:** 1 Indiana University, Indianapolis, Indiana, United States; 2 Pennsylvania State University, University Park, Pennsylvania, United States; 3 Penn State, University Park, Pennsylvania, United States

## Abstract

Adults aged 50 and older are at risk for human immunodeficiency virus (HIV) infection. Currently, there are no measures specifically aimed at middle-aged and older adults to assess their HIV risk. Existing measures have been created for and mostly tested in adolescent and young adult populations. The purpose of this study was to modify and test existing instruments related to HIV prevention factors with an older adult population. Two rounds of an expert panel (N = 10) review were conducted to assess items from the Condom Use Self-Efficacy Scale and the Sexual Risks Scale for their applicability to older adults. Any items with content validity at the item level <0.78 were either discarded or modified. New items were also added. The final adapted HIV prevention scale had 31 items and was administered via an online survey. Single adults (N = 252) aged 50 to 85 who had been on at least one date over the past year participated in the study. The HIV prevention scale underwent confirmatory factor analysis. Model fit was estimated using maximum likelihood and standardized estimates were used for factor loadings. The items loaded on eight factors in three models: Model 1 (Mechanics, Advocacy, Intoxicants); Model 2 (Attitudes, Normative Beliefs, Perceived Susceptibility); and Model 3 (Intention, Expectations). There was adequate to excellent model fit. However, there were multiple correlations of error variances suggesting that while the items are appropriate for an older adult population, the scale will need adaptations prior to using for further data collection.

